# Culinary Acculturation Among International Students in Türkiye: Behavioral Insights and the Development of an AI-Supported Interactive Platform

**DOI:** 10.3390/bs16050667

**Published:** 2026-04-28

**Authors:** Merve Çapaş, Betül Çiçek, Kübra Minyas, Rahma Mahnoor

**Affiliations:** 1 Department of Nutrition and Dietetics, Faculty of Health Sciences, Erciyes University, Kayseri 38039, Türkiye; bcicek@erciyes.edu.tr; 2 Department of Biochemistry, Faculty of Pharmacy, Gazi University, Ankara 06330, Türkiye; kubraminyas@gmail.com; 3 Department of Theoretical and Translational Medicine, Faculty of Medicine, Semmelweis University, Budapest 1085, Hungary; rahma.mahnoor@phd.semmelweis.hu

**Keywords:** culinary acculturation, eating behaviors, international students, Turkish cuisine, food choices, cultural integration, AI-supported platform

## Abstract

This study investigated the adaptation of culinary culture and behavioral adjustment to Turkish cuisine among international students. The sample comprised 82 students (61.0% males; 39.0% females) from over 20 countries across Europe, Central Asia, South/Southeast Asia, Africa, and the Middle East, all enrolled at Erciyes University. Data collection involved a sociodemographic questionnaire, assessments of food consumption frequency and cooking methods, and the Culinary Culture Adaptation Assessment Inventory. Results indicate that adaptation to Turkish cuisine occurs through a selective and gradual behavioral process. Higher adaptation levels were observed for basic dietary components (bread, soup, rice, yoghurt, and tea), whereas adoption of starch- and sugar-heavy dietary patterns was more limited. Gender comparisons revealed significantly higher scores for meat-heavy and starch-heavy dietary patterns among males (*p* = 0.048 and *p* = 0.031, respectively). In contrast, regional origin, economic status, and language proficiency were not significantly associated with culinary acculturation levels. Comparisons based on length of residence identified significant differences in meat-heavy and starch-heavy dietary patterns (*p* = 0.034 and *p* = 0.008, respectively). Cooking behaviors remained stable for boiling, grilling, and baking, while frying and roasting decreased. Reported changes in portion perception and body weight suggest that culinary acculturation may extend beyond food choice to broader eating behaviors. Based on these results, an AI-supported interactive platform was developed to facilitate culturally comparable food matching between Turkish and global cuisines. These findings may inform the development of culturally sensitive strategies to support culinary adaptation among international students.

## 1. Introduction

The internationalization of higher education has reached unprecedented levels, reshaping the demographic and cultural landscapes of host countries worldwide. Türkiye has become a major destination for internationally mobile students due to its strategic location, cultural diversity, and rich culinary heritage (Çetin et al., 2017; Yalçın, 2023). According to official statistics, the number of international students in Türkiye increased from 43,251 in 2013 to 301,694 in 2023, positioning the country among the leading global hosts of international students (Council of Higher Education (YÖK), 2023). This rapid demographic transformation has intensified scholarly interest in the multi-dimensional adaptation processes experienced by international students (Demir et al., 2025).

Acculturation is the term used to describe the psychological and behavioral transformations that occur when individuals meet a new cultural context. Although prior research has largely focused on academic integration, language acquisition, and social adjustment (Erturk & Luu, 2022; Ünsal et al., 2024), food-related behaviors represent one of the most visible yet comparatively underexplored domains of everyday acculturation. Culinary practices function simultaneously as markers of cultural identity and as sites of negotiation between heritage traditions and host-country norms. Within this context, international students may develop hybrid dietary patterns that combine familiar tastes with local culinary elements (Sali, 2023). However, adaptation is rarely linear. Experiences of uncertainty and disruption conceptualized in the literature as “gastro-anomie” may reshape eating behaviors in complex ways. Additionally, food neophobia and food insecurity have been identified as behavioral barriers that may influence dietary decision-making during cultural transition (Bellikci-Koyu et al., 2024; Sözlü et al., 2025).

Existing studies have explored international students’ attitudes toward Turkish cuisine (Ozgen & Yaman, 2014), the role of cultural familiarity in shaping culinary intentions (Şahin & Cömert, 2025), intercultural competence (Aksezer et al., 2022), and gastrodiplomatic perspectives (Aslan, 2025; Hussein et al., 2024). Although these contributions provide valuable descriptive insights, relatively few studies have simultaneously examined dietary patterns, cooking behaviors, and portion-related changes using validated measurement tools. The Culinary Acculturation Assessment Inventory (CAAI) offers a structured framework for assessing such behavioral dimensions (Kalyoncu et al., 2021); however, its application among heterogeneous international student populations in Türkiye remains limited.

In this context, the notion of “selective culinary acculturation” is introduced as an interpretive lens to describe the uneven and domain-specific nature of culinary acculturation, whereby certain food-related practices are more readily adopted than others. Rather than representing a distinct theoretical model, this concept is used to characterize observable patterns within culinary adaptation processes and to complement existing dietary acculturation frameworks. Furthermore, while previous research has predominantly relied on survey-based analyses, the present study complements preliminary findings by introducing a conceptual digital platform (CulinaryVerse). This platform is not empirically evaluated within the scope of the current study and is introduced as a conceptual application derived from observed behavioral patterns. During the analysis, students’ culinary knowledge appeared to be associated with differences in culinary acculturation patterns. Based on this observation, a digital platform was conceptually developed to explore potential matches between traditional dishes from different cuisines and Turkish cuisine. The platform is intended to illustrate how behavioral insights may inform future research and potential applications in culinary acculturation.

To our knowledge, no previous study has simultaneously examined culinary acculturation patterns, cooking behaviors, and portion-related changes using a structured instrument such as the CAAI within a heterogeneous international student population in Türkiye. Therefore, this study addresses an important gap in literature by providing a multidimensional behavioral assessment of culinary acculturation in this context.

Given the scale of international student mobility and the central role of food practices in daily life, a comprehensive, multi-dimensional investigation of culinary acculturation is necessary. This study analyzes the behavioral patterns of culinary acculturation among international students at Erciyes University by examining changes in dietary preferences, cooking methods, and portion perceptions using the CAAI framework.

Accordingly, the study examines patterns of culinary acculturation across dietary, cooking, and portion-related domains, with a focus on whether these patterns reflect a selective and non-linear process.

As an exploratory study, the findings are intended to provide initial empirical insights into culinary acculturation processes rather than to establish causal relationships or evaluate intervention effects.

## 2. Materials and Methods

### 2.1. Study Design and Participants

This cross-sectional descriptive study examined the culinary acculturation patterns and behavioral dietary changes among international students enrolled at Erciyes University in Türkiye. The study was designed as an exploratory quantitative investigation aimed at identifying patterns of culinary adaptation rather than testing causal relationships. As an exploratory study, the primary aim was to identify patterns and generate initial insights rather than to achieve statistical representativeness or test predefined hypotheses. A criterion-based purposive sampling strategy recruited participants through international student networks within the university, and voluntary participation. Students who met pre-defined eligibility criteria and agreed to participate were included. Therefore, the sample reflects an accessible and heterogeneous subgroup of international students rather than a statistically representative population. In total, 82 international students completed the survey. The sample size was not determined through a formal priori calculation but was based on the accessible population of international students who met the inclusion criteria and agreed to participate during the study period.

Inclusion criteria were being a non-Turkish citizen, residing in Türkiye for at least two months, being between 18 and 35 years of age, having no communication impediment, and possessing sufficient English proficiency to complete the questionnaire. Exclusion criteria included having a physician-diagnosed metabolic disorder, following a medically prescribed special diet, presenting cognitive impairment, or being unable to provide informed responses.

Structured questionnaires were administered in person to gather data. Face-to-face data collection was preferred to enhance response accuracy and to allow clarification of culturally specific food-related items when necessary. Each interview lasted around 20 min, and participants were contacted in advance to schedule meetings. Written and verbally informed consent was obtained before participation, and anonymity and confidentiality were ensured throughout the research process. Participation was voluntary, and participants were informed of their right to withdraw at any stage without consequence. No incentives were provided.

The survey instrument used in this study was subsequently embedded in a digital platform (CulinaryVerse) to support future data collection; however, this integration was not part of the empirical data collection process of the present study.

### 2.2. Measures

#### 2.2.1. Structured Questionnaire

Data was collected via a structured questionnaire consisting of five sections.

The first section gathered sociodemographic information, including age, gender, body weight, height, education level and duration, occupation, marital status, self-perceived economic status, presence of diagnosed chronic diseases, physical activity characteristics (type, frequency, duration), and alcohol consumption.

The second section assessed the frequency of consumption of Turkish cuisine-specific foods by an eight-point Likert-type scale ranging from “every meal” to “never consumed.” Food items included bread, pastry products, rice and bulgur dishes, soups, kebab/pide/lahmacun, meat and vegetable stews, meatballs, processed meats (e.g., sausage, pastrami), offal, olive oil-based vegetable dishes, salads, pickles, salad dressings, seasonal fruits and vegetables, traditional Turkish breakfast components, yoghurt/ayran/kefir, spices, milk-based desserts, syrup-based desserts, fruit desserts, confectionery, black tea, Turkish coffee, and local beverages.

The third section collected migration-related and cultural variables, including region of origin, length of stay in Türkiye, Turkish language proficiency, primary news sources, presence of Turkish individuals within the nuclear family, cohabitation with Turkish individuals, and property ownership in Türkiye.

The fourth section evaluated perceived changes following relocation to Türkiye in body weight, portion size, cooking methods, and food consumption patterns by a five-point Likert-type scale ranging from “much less” to “much more.” Cooking methods assessed included low-oil frying, deep-frying, grilling, baking, boiling, and microwave cooking. Changes in food and beverage consumption were assessed for vegetables, potatoes, rice, snacks, fruits, sugar-sweetened beverages, dairy products, fried foods, desserts, confectionery, white meat, and red meat.

Furthermore, food preparation and eating behaviors (e.g., preparing Turkish breakfast, cooking traditional Turkish meals, using sunflower oil, olive oil, or butter, preparing tomato paste-based dishes, producing traditional preserved foods, eating meals with family or friends, aligning meal timing with Turkish norms, consuming three main meals daily, and eating at a traditional floor table) were evaluated by a seven-point frequency scale.

#### 2.2.2. Culinary Acculturation Assessment Inventory (CAAI)

Culinary acculturation patterns were assessed using the CAAI, a validated 35-item instrument developed by Kalyoncu et al. (2021). The scale comprises five sub-patterns reflecting different dimensions of culinary adaptation.

The basic dietary pattern includes staple daily foods such as bread, soup, hot dishes, olive oil-based dishes, vegetables, breakfast items, yoghurt, coffee, and tea. The meat-oriented pattern consists of rice-based dishes consumed with meat, kebabs, meatballs, processed meat, and offal. The starch-heavy pattern evaluates carbohydrate- and dessert-dominant items, including simit, milk-based and syrup-based desserts, ashura, fruit desserts, and confectionery products. The accessory foods pattern includes side dishes accompanying the main meals, such as appetizers (meze), pickles, salad dressings, and compotes. The culinary practices pattern assesses food preparation and cooking behaviors.

For each sub-pattern, mean scores were calculated and subsequently standardized by using a z-score transformation to ensure comparability across patterns. The CAAI does not yield a global total score; therefore, sub-pattern scores were analyzed independently. The original validation study demonstrated satisfactory reliability and validity for the scale (Kalyoncu et al., 2021). In the present study, internal consistency reliability was assessed using Cronbach’s alpha coefficients for each sub-pattern.

### 2.3. Statistical Analysis

All statistical analyses were performed using SPSS version 22.0 (IBM Corp., Armonk, NY, USA). Continuous variables were presented as mean ± standard deviation, while categorical variables were expressed as frequency and percentage. Normality assumptions were evaluated using the Shapiro–Wilk test. Homogeneity of variances was assessed using Levene’s test. Independent samples *t*-tests were used for two-group comparisons. One-way analysis of variance (ANOVA) was applied for comparisons involving three or more groups, and Welch-adjusted ANOVA results were reported when the homogeneity assumption was violated. Post hoc pairwise comparisons were conducted when necessary. Associations between categorical variables were evaluated using chi-square (χ^2^) tests. Statistical significance was set at *p* < 0.05. In addition to *p*-values, effect sizes were reported to provide an estimate of the magnitude of group differences. Cohen’s d was used for independent samples *t*-tests, and eta squared (η^2^) was reported for ANOVA analyses. Given the exploratory design and the available sample size, the analyses were considered sufficiently sensitive to detect moderate effect sizes, while smaller effects may not have been fully captured. Therefore, results were interpreted with caution, with emphasis placed on overall patterns rather than isolated statistically significant findings.

### 2.4. Development of the AI-Supported Interactive Platform

The platform was not empirically evaluated within the scope of this study and is presented as a conceptual and applied extension of the findings. Drawing on the study’s empirical findings, an AI-supported interactive web platform (CulinaryVerse; https://culinaryverse.com) was developed as an applied outcome of the research. The system was created to assist in matching culturally related foods from Turkish cuisine with those of other cuisines to facilitate culinary adaptation. The platform architecture consists of a PHP-based front-end user interface, a PHP-based backend processing layer for data handling and request management, and a MongoDB database for flexible, scalable data storage. The system allows users to select Turkish dishes and identify culturally similar alternatives from global cuisines, presenting structured recipe-based matching as a potential tool for exploring culturally comparable foods across cuisines.

In addition, the platform integrates the survey instrument used in the present study, enabling international students to participate online and supporting the continuous accumulation of data for future research on culinary acculturation.

### 2.5. Ethical Approval

This study was conducted in accordance with the Declaration of Helsinki and approved by the Erciyes University Social and Human Sciences Ethics Committee (Approval No: 91, dated 27 February 2024). Written informed consent was obtained from all participants prior to data collection. All procedures ensured confidentiality and compliance with ethical standards for research involving human participants.

### 2.6. Generative Artificial Intelligence Disclosure

No generative AI tools were used in the design, data collection, statistical analysis, or interpretation of this study. The web platform described is a digital application derived from the study findings. It does not perform automated data analysis or generate research outputs for this manuscript.

## 3. Results

### 3.1. Participant Characteristics

Eighty-two international students participated. The mean age was 23.24 ± 4.03 years, and the mean body weight and height were 67.02 ± 13.64 kg and 170.7 ± 10.68 cm, respectively.

Participants represented diverse demographic and geographic backgrounds, reflecting the multicultural composition of the university’s international student population. The bulk of students had been residing in Türkiye for less than one year. Participants originated from multiple regions, including the Middle East (31.6%), Africa (28.9%), South/Southeast Asia (18.4%), Central Asia (10.5%), and Europe (10.5%), reflecting a culturally heterogeneous sample. A detailed overview of the participants’ sociodemographic and anthropometric characteristics is presented in Table 1.

### 3.2. Interaction with Turkish Culinary Practices

International students reported varying levels of engagement with Turkish culinary practices in their daily routines. A considerable proportion of participants indicated that they regularly incorporated Turkish cuisine into their weekly meal plans; 40.3% reported Turkish dish consumption for two to three times per week, and an additional 34.1% reported doing so daily. Similarly, shared meal practices were common, with 40.3% of students reporting eating together with others several times per week.

It appeared that direct preparation of traditional Turkish dishes was less frequent. Approximately one-third of participants (34.1%) reported never cooking Turkish dishes themselves. Preparing Turkish breakfast and traditional beverages, such as tea, was also reported less frequently, with many students indicating they prepared these items only occasionally.

In contrast, certain structural elements of Turkish cuisine were more readily integrated into everyday cooking practices. For example, the use of oil in cooking was reported as a daily practice by 34.1% of the participants, while the use of tomato paste, an essential component of many Turkish dishes, was reported by 34.2% of students at least for two to three times per week.

These findings suggest that engagement with Turkish cuisine may occur more frequently through the use of common ingredients and routine practices than through direct preparation of traditional dishes. Detailed distributions are described in Supplementary Table S1.

### 3.3. Frequency of Turkish Food Consumption

The reported frequency of Turkish food consumption showed clear differences across food groups, with staple items being consumed substantially more frequently than culturally distinctive foods. Bread and grain-based foods, such as rice and bulgur, were among the most frequently consumed items; for example, 56.1% of the participants reported consuming bread daily, while 86.5% reported consuming rice, bulgur, or pasta at least several times per week.

Traditional Turkish dishes, including soups, kebabs, and stews, were also commonly consumed weekly. Soup was reported to be consumed weekly or daily by 78.1% of the participants. In comparison, kebab or döner-type dishes were consumed at least weekly by 58.6% of the students. These patterns indicate frequent exposure to widely available food items within Turkish cuisine.

In contrast, certain culturally distinctive foods, such as simit, pickles, and some traditional desserts, were consumed less frequently and were generally reported as occasional or monthly items. Among beverages, tea was the most frequently consumed drink, with 58.5% of participants reporting daily consumption, whereas fermented dairy beverages, such as ayran and kefir, were also relatively common, with 76.8% of students reporting consumption at least several times per week. The full distribution of consumption frequencies for Turkish foods is provided in Supplementary Table S2.

### 3.4. Changes in Food Consumption After Arrival in Türkiye

Changes in food consumption patterns were examined to evaluate how international students’ dietary habits evolved during their stay in Türkiye. These changes were assessed using a five-point response scale ranging from “much less” to “much more”. Overall, responses tended to cluster around “no change” and “more” categories across most food groups indicating generally modest changes in reported dietary patterns. Table 2 presents the distribution of self-reported changes in the consumption of selected food groups following arrival in Türkiye.

Increases were particularly noticeable for vegetables, rice, and snack foods, suggesting that the availability and diversity of foods in Türkiye may have expanded the participants’ individual food choices. In contrast, participants reported higher proportions of less red meat and potato consumption. These patterns may be associated with multiple contextual factors, although no causal inference can be made.

Fruit consumption showed a relatively unchanged pattern, with most participants reporting either no change or more intake. Only a small proportion reported reduced fruit consumption, suggesting that fruit is a culturally neutral and widely accepted food group across different dietary traditions. Regional comparisons did not reveal significant variations in changes in fruit consumption (*p* > 0.05).

For soft drinks, a large proportion of participants reported either no change or reduced consumption. It is important to note that some students indicated substantially lower consumption levels after arrival in Türkiye. Nevertheless, these changes did not differ across geographical regions.

Dairy product consumption showed a relatively balanced distribution, with many participants reporting no change in intake and a considerable proportion reporting in-creased intake. The prominence of yogurt and fermented dairy products in Turkish cuisine may partly explain this pattern. Similarly, changes in dairy consumption did not differ by region.

There was a decrease in the consumption of fatty and fried foods among the subjects. This pattern is comparable to the observed changes in cooking techniques reported in other sections of the study. Nevertheless, regional comparisons once more failed to reveal any substantial disparities. For sweets and confectionery products, many participants reported either unchanged or increased consumption. The diversity of desserts in Turkish cuisine may have contributed to this increase. Likewise, white meat consumption displayed a pattern characterized mainly by unchanged or increased intake. In both cases, regional differences were not significant.

Overall, no statistically significant regional differences were observed across food groups (*p* > 0.05). However, changes in food consumption appear more closely associated with the local food environment, culinary culture, and daily dietary practices in Türkiye.

### 3.5. Changes in Cooking Methods

Participants reported several changes in their food preparation practices after relocating to Türkiye. The distribution of perceived changes in cooking methods is presented in Table 3. Changes in cooking practices during students’ stay in Türkiye were evaluated across various preparation techniques. Across most cooking methods, responses were concentrated in the “no change” and “less” categories, indicating relatively stable or reduced use of certain cooking techniques.

For stir-frying, 48.7% of participants reported using this method less or much less frequently, whereas 32.9% indicated no change. A similar pattern was observed in the case of deep-frying: 47.4% reported reduced use and 35.5% reported no change in frequency. A decrease in the reported use of oil-intensive cooking methods was noted.

For grilling and baking, decreases were also reported, although the “no change” category became more prominent. In grilling, 38.2% of the participants reported no change, while 51.3% indicated less use. For baking, 31.6% reported unchanged usage levels.

Boiling and microwave cooking showed different patterns. For boiling, 44.7% of the participants reported no change, while 21.1% reported more use. In the same vein, microwave cooking was largely stable, with 47.4% reporting no change, and increases and decreases distributed more evenly across participants.

Regional comparisons using Chi-square analysis indicated that changes in cooking methods did not differ significantly across regions. No significant regional differences were observed for stir-frying (χ^2^ = 9.06; *p* = 0.911), deep-frying (χ^2^ = 18.60; *p* = 0.290), grilling (χ^2^ = 23.04; *p* = 0.113), baking (χ^2^ = 13.37; *p* = 0.646), boiling (χ^2^ = 12.39; *p* = 0.717), or microwave cooking (χ^2^ = 16.57; *p* = 0.414). These findings did not differ significantly across regions.

### 3.6. Changes in Body Weight and Portion Size Perceptions

Changes in body weight and portion size perceptions were examined to characterize behavioral aspects of culinary acculturation among international students during their stay in Türkiye. Regarding body weight changes, 36.6% of participants reported weight loss during the previous year, while 23.2% reported weight gain. Another 23.2% a stable body weight. According to perceptions of portion size, nearly half (47.6%) reported no change in portion size after relocating to Türkiye. However, 29.2% of students reported consuming smaller portions, whereas 23.1% reported consuming larger portions. These findings describe variation in perceived body weight and portion size following relocation.

### 3.7. Culinary Acculturation Patterns (CAAI) and Associated Sociodemographic Factors

Internal consistency reliability of the CAAI subdimensions was assessed using Cronbach’s alpha coefficients. The basic dietary pattern showed acceptable reliability (α = 0.71), while the meat-oriented pattern demonstrated moderate reliability (α = 0.64). The starch-heavy (α = 0.80), accessory foods (α = 0.76), and culinary practices (α = 0.77) patterns showed acceptable to good internal consistency. These results indicate that the scale performed with generally acceptable reliability across subdimensions in the present sample.

Culinary acculturation among 82 international students was assessed using the five subdimensions of the CAAI: basic dietary pattern, meat-oriented dietary pattern, starch-heavy dietary pattern, accessory foods pattern, and culinary practices pattern (Table 4). The highest mean score was observed for the basic dietary pattern (5.33 ± 1.06), indicating higher scores for staple-related items in the participants’ daily dietary routines. In contrast, the starch-heavy dietary pattern exerted the lowest mean score (3.96 ± 1.19), indicating lower relative scores in this domain. Intermediate scores were recorded for the meat-oriented dietary pattern (4.38 ± 1.08), the accessory foods pattern (4.46 ± 1.52), and the culinary practices pattern (4.40 ± 1.25), reflecting the moderate levels of adaptation in these domains.

Gender differences were evident in selected culinary acculturation patterns. Male participants scored significantly higher than females in the meat-oriented dietary pattern (4.57 ± 1.14 vs. 4.09 ± 0.93; *p* = 0.048; Cohen’s d ≈ 0.45) and the starch-heavy dietary pattern (4.18 ± 1.33 vs. 3.61 ± 0.84; *p* = 0.031; Cohen’s d ≈ 0.49). No significant gender differences were observed for the basic dietary pattern, accessory foods pattern, or culinary practices pattern.

Duration of residence in Türkiye was selectively associated with culinary acculturation. While the basic dietary, accessory foods, and culinary practices patterns remained stable across residence duration groups (≤12 months, 13–36 months, >36 months), significant differences emerged for the meat-oriented (*p* = 0.034; η^2^ ≈ 0.08)and starch-heavy (*p* = 0.008; η^2^ ≈ 0.08) dietary patterns. These findings indicate variation across residence duration groups, without implying a causal or directional relationship, and correspond to moderate effect sizes. Living with a Turkish individual was also associated with variation in culinary acculturation. Students who shared with a Turkish individual had significantly lower scores in the starch-heavy dietary pattern (*p* = 0.022), whereas no significant differences were observed for the remaining CAAI dimensions.

In contrast, several other sociodemographic factors showed no significant association with culinary acculturation patterns. No significant differences were identified across geographical regions of origin (Africa, Central Asia, Europe, the Middle East, and South/Southeast Asia), perceived economic status, language proficiency, or general social contact.

Across the main comparative analyses, effect size estimates indicated that the study was capable of detecting moderate group differences. However, comparisons involving multiple or unevenly distributed subgroups (e.g., region or language proficiency) were generally associated with small effect sizes (η^2^ < 0.06), suggesting limited sensitivity for detecting subtle differences in these analyses.

Variation across sociodemographic groups was concentrated primarily in the meat-oriented and starch-heavy dietary patterns, whereas the basic dietary pattern and culinary practices remained relatively stable. Overall, variation across subdimensions was limited, with statistically significant differences observed only in selected domains. Culinary acculturation appears largely regardless of most background characteristics, while gender, length of stay, and close household-level cultural exposure may influence specific dietary patterns. The observed pattern further suggests that the staple elements of the host cuisine are incorporated more readily, whereas culturally distinctive foods require longer or more context-dependent exposure, and that adaptation may be shaped more strongly by the local food environment encountered in Türkiye than by the students’ original cultural background.

## 4. Discussion

**Figure 1:**
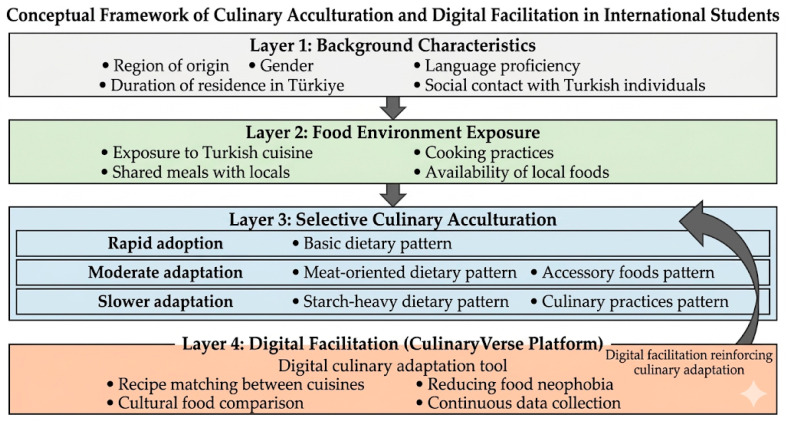
Conceptual framework linking food environment exposure, selective culinary acculturation patterns, and digital facilitation through the CulinaryVerse platform.

The present study examined culinary acculturation among international students in Türkiye and revealed a patterned process of adaptation that appears to be shaped by exposure to the local food environment and daily dietary practices. Consistent with the empirical patterns observed in the CAAI subdimensions, the results indicate that culinary acculturation does not occur uniformly across dietary domains and may be characterized as a selective process, in which staple components of the host cuisine are incorporated relatively rapidly while more culturally distinctive foods and specific culinary practices are adopted more gradually. In particular, the relatively high scores observed in the basic dietary pattern suggest that widely accessible and structurally central elements of Turkish cuisine, such as bread, soups, vegetables, yoghurt, and tea, are integrated into students’ daily diets with relative ease. In contrast, carbohydrate-rich traditional foods and certain cooking practices may require longer exposure and familiarity before being incorporated into routine consumption patterns. This asymmetrical pattern of adoption indicates that culinary acculturation may be shaped by both cultural background and repeated exposure to the host-country food environment. Taken together, these findings point to a non-uniform and gradual process of adaptation, rather than a complete or uniform shift toward host-country dietary norms. Overall, the findings describe a gradual and domain-specific pattern of culinary acculturation rather than a uniform dietary transition (see Figure 1).

Similar patterns of gradual culinary acculturation in food-related behaviors have been described in studies examining the dietary acculturation among internationally mobile populations, in which exposure to the host environment shapes daily food choices and eating practices (Eluwole et al., 2020). This pattern also aligns with the concept of selective acculturation, which posits that individuals tend to adopt certain elements of the host culture while retaining aspects of their original cultural practices (Dean et al., 2024). The present findings are consistent with existing literature highlighting the role of environmental exposure and selective adaptation processes in shaping culinary acculturation. Overall, the study provides descriptive evidence on how culinary acculturation may unfold across different domains among international students.

The selective nature of culinary adaptation observed in this study suggests a layered pattern in the integration of host-country food practices, rather than a uniform transformation of dietary behavior. Staple components of the host cuisine, particularly those that are widely available and easily incorporated into daily meals, appear to be adopted more rapidly. In contrast, culturally distinctive or preparation-intensive foods associated with specific culinary traditions tend to require longer exposure before being incorporated into routine consumption patterns. This layered pattern of adoption may be associated with the complex interaction between familiarity, accessibility, and experiential learning within the host-country food environment. Over time, repeated exposure to common ingredients, shared meals, and daily eating may be linked to gradual changes in students’ culinary repertoire, while elements of their original dietary habits are maintained.

Previous research suggests that participatory culinary activities, such as cooking classes, may function as experiential learning environments through which individuals engage more deeply with the cultural meanings of food practices (Yiğit, 2022). Research examining food-related behaviors among visitors and international populations similarly indicates that exposure to diverse food environments and individual levels of food involvement may influence willingness to try unfamiliar foods and shape dietary exploration (Derinalp Çanakçı & Birdir, 2020). This interpretation also resonates with the “loyal tongue, liberal mind” perspective, suggesting that individuals may cognitively adapt to a new cultural context while maintaining relatively stable taste preferences and established dietary habits (O’Sullivan & Amirabdollahian, 2016).

The results also highlight the potentially important role of the host-country food environment in shaping culinary adaptation among international students. Daily exposure to locally available foods, shared meals with peers or residents, and participation in routine eating contexts may contribute to familiarity with the host cuisine. Such environmental exposure may increase opportunities to encounter local ingredients and may normalize certain eating practices within daily routines. Students in the present study also reported decreases in body weight and a reduced preference for oil-intensive cooking techniques such as frying, which may be associated with contextual changes in the surrounding food environment. Traditional home-style meals commonly available in Turkish university settings may be associated with differences in dietary practices. This observation contrasts with results from studies conducted in Western host countries, where international students have sometimes experienced weight gain associated with increased accessibility to fast-food environments (Alakaam & Willyard, 2020; Shetty et al., 2024). Over time, these daily experiences may be associated with reduced uncertainty and increased willingness to try local dishes, ultimately contributing to changes in dietary repertoire. Similar observations have been reported in research on international students’ experiences in host societies, where daily interactions and environmental exposure contribute to broader processes of cultural and behavioral adaptation (Wu et al., 2015). Campus dining environments and institutional food systems have also been identified as important contextual factors shaping the international students’ dietary experiences and daily food choices during their adaptation processes (Corcoran, 2018).

The examination of sociodemographic variables provides additional insight into the factors that influence culinary adaptation among international students. Gender differences observed in the meat-oriented and starch-heavy dietary patterns suggest that certain food preferences may be influenced by gender-related consumption tendencies or cultural expectations associated with meal composition. Research has demonstrated that culturally embedded gender stereotypes may shape food preferences and consumption intentions, particularly regarding meat-based foods often associated with masculinity (Ekebas-Turedi et al., 2021). Conversely, several other variables including regions of origin, self-perceived economic status, language competence, and overall social contact were not significantly associated with culinary acculturation patterns.

Studies focusing on international students’ eating behaviors have also suggested that concerns related to dietary stress, body weight, and health awareness may influence food choices during cultural transition (Jin et al., 2023; Yurochko et al., 2021). These findings suggest that culinary acculturation may not be strongly associated with cultural background or socioeconomic characteristics within this sample. Although cultural distance has been identified as an important factor influencing the international students’ broader adaptation processes (Aydın & Örs, 2025), the present results suggest that culinary acculturation may be more closely associated with daily food environments than with perceived cultural differences between the host and home countries. In this context, daily exposure to the host-country food environment may play a more prominent role in shaping dietary integration.

The length of stay in Türkiye may also appear to play a selective role in shaping certain aspects of gastronomic adaptation. Significant differences observed in the meat-oriented and starch-heavy dietary patterns across residence duration groups suggest that familiarity with specific local dishes may develop gradually with prolonged exposure to the host-country food environment. Over time, repeated encounters with local ingredients, cooking styles, and dining environments at restaurants or on campus may be associated with increased willingness to incorporate Turkish foods into daily diets. In addition to temporal exposure, household-level interaction with local individuals may represent another important pathway for culinary adaptation. Studies focusing on the international students’ daily experiences in host societies have also highlighted that communication barriers and cultural differences may influence daily interactions, shaping how individuals navigate services, food environments, and social contexts (Demirel & Serbes, 2023). Previous cross-cultural research has also reported that international students often encounter various intercultural challenges during their adaptation, including differences in communication styles, social expectations, and daily practices within the host society (Cetindere & Shin, 2024).

Close social interactions may offer practical opportunities to observe food preparation practices, experience local meal structures, and become familiar with culturally embedded eating routines. Household-level interactions may also support observational learning and facilitate gradual adaptation to local dietary routines within shared living environments (Shetty et al., 2024). These interactions may be associated with reduced uncertainty in unfamiliar food environments, a phenomenon conceptualized in the literature as “gastro-anomie.” They may contribute to the emergence of hybrid dietary identities in which individuals maintain elements of their original culinary traditions while incorporating practices from the host culture (Gebru & Yuksel-Kaptanoglu, 2020). Together, these results suggest that both duration of exposure and the intensity of daily social interaction may shape the trajectory of culinary acculturation.

Beyond its several contributions, the present study provides preliminary insights into how behavioral patterns identified in this research may inform potential applications. Drawing on these insights, CulinaryVerse is introduced as a conceptual tool for exploring culinary similarities and differences across contexts. By enabling the identification of similarities between dishes from different cuisines, the platform may offer a structured way to explore unfamiliar foods. Such tools may be relevant in addressing barriers such as food neophobia and limited familiarity with host-country culinary traditions. In this context, CulinaryVerse may be considered as an informational resource that illustrates how digital tools can support engagement with local food practices while maintaining connections with familiar dietary elements.

The integration of preliminary findings into a digital platform illustrates how empirical insights into culinary acculturation may be translated into potential applications in multicultural educational settings. From a conceptual perspective, such tools may relate to digitally mediated food exploration and support navigation of unfamiliar food environments. These observations may have implications for universities hosting international students, particularly in relation to culturally informed food guidance within student support programs.

Several limitations of this investigation should be acknowledged. First, the research used a cross-sectional design, which limits the ability to infer causal relationships between exposure to the host-country food environment and changes in culinary behavior. Second, the sample was drawn from a single university and consisted of a relatively small number of participants, which may limit the generalizability of the results to other international student populations. Additionally, the sample was based on a convenience sampling approach rather than a probability-based design, which may further limit representativeness. In addition, the data relied on self-reported responses, which may be susceptible to recall bias or social desirability effects, potentially affecting the accuracy of reported dietary behaviors. Furthermore, although the sample size allowed the detection of moderate effect sizes in key comparative analyses, the study may have been underpowered to detect small differences, particularly in analyses involving multiple or unevenly distributed subgroups (e.g., region or language proficiency). Despite these limitations, the study provides descriptive insights into the behavioral dimensions of culinary acculturation among internationally mobile students and offers an initial empirical basis for understanding how daily food environments may be associated with the culinary acculturation processes. Although the results are based on a single institutional context, they may provide context-specific insights for comparable higher education settings.

Future research may facilitate understanding of culinary acculturation processes by incorporating larger, more diverse international student populations across multiple universities and cultural contexts. Longitudinal research designs may also offer useful insights into how culinary adaptation evolves as students gain prolonged exposure to the host-country food environment. In addition, qualitative or mixed-methods approaches may provide deeper insight into the experiential and contextual dimensions of culinary adaptation that cannot be fully captured through quantitative measures alone. In this regard, the CulinaryVerse digital platform developed in this study may serve as a conceptual framework for future applications, although this requires empirical validation. Such platforms may support future data collection and cross-cultural comparisons of culinary acculturation patterns. Future studies may also investigate the endorsement related to the digitally supported tools designed to reduce food-related uncertainty, which are associated with culinary exploration and culinary acculturation among international students.

## 5. Conclusions

This study examined the culinary acculturation among international students in Türkiye and suggests that adaptation to the host-country’s food environment may occur in a selective, multi-layered manner. The results suggest that staple and widely accessible components of Turkish cuisine are incorporated into students’ nutritional behaviors relatively rapidly, whereas culturally distinctive foods and certain culinary practices tend to be adopted more gradually.

These results highlight the potential role of daily food environments and exposure to local cuisine in shaping culinary acculturation among international students. The findings provide descriptive evidence supporting the notion of selective culinary acculturation within this study context. In addition, the CulinaryVerse digital platform is introduced as a conceptual extension illustrating how behavioral insights may inform potential future applications. Taken together, the findings suggest that behavioral insights may inform future research and support strategies related to culinary acculturation in international higher education settings.

## Figures and Tables

**Table 1:** Socio-demographic characteristics of international students (n = 82).

	Variable	n (%)
Gender	Male	50 (61.0)
	Female	32 (39.0)
Economic Status	Income less than expenses	33 (40.2)
(self-reported)	Income equals expenses	40 (48.8)
	Income exceeds expenses	9 (11.0)
Length of stay in Türkiye	≤12	58 (70.7)
(months)	13–36	12 (14.6)
	>36	12 (14.6)

**Table 2:** Changes in food consumption patterns after arrival in Türkiye (n = 82).

Level of Change in Food Consumption	Africa n (%)	Central Asia n (%)	Europe n (%)	Middle East n (%)	South/South East Asia n (%)	*p*
VEGETABLES						
Much less	2 (9.1)	0 (0.0)	2 (25.0)	5 (20.8)	3 (21.4)	0.556
Less	5 (22.7)	1 (12.5)	0 (0.0)	5 (20.8)	2 (14.3)	
No change	7 (31.8)	3 (37.5)	5 (62.5)	10 (41.7)	3 (21.4)	
More	7 (31.8)	3 (37.5)	1 (12.5)	4 (16.7)	4 (28.6)	
Much more	1 (4.6)	1 (12.5)	0 (0.0)	0 (0.0)	2 (14.3)	
POTATOES						
Much less	4 (18.2)	0 (0.0)	0 (0.0)	2 (8.3)	1 (7.1)	0.785
Less	3 (13.6)	1 (12.5)	2 (25.0)	6 (25.0)	3 (21.4)	
No change	7 (31.8)	3 (37.5)	5 (62.5)	9 (37.5)	6 (42.9)	
More	4 (18.2)	3 (37.5)	0 (0.0)	6 (25.0)	3 (21.4)	
Much more	4 (18.2)	1 (12.5)	1 (12.5)	1 (4.2)	1 (7.1)	
RICE						
Much less	2 (9.1)	0 (0.0)	0 (0.0)	3 (12.5)	3 (21.4)	0.151
Less	2 (9.1)	0 (0.0)	1 (12.5)	8 (33.3)	5 (35.7)	
No change	7 (31.8)	2 (25.0)	3 (37.5)	5 (20.8)	2 (14.3)	
More	8 (36.4)	5 (62.5)	4 (50.0)	4 (16.7)	1 (7.1)	
Much more	3 (13.6)	1 (12.5)	0 (0.0)	4 (16.7)	3 (21.4)	
SNACKS						
Much less	2 (9.1)	1 (12.5)	1 (12.5)	2 (8.3)	1 (7.1)	0.315
Less	5 (22.7)	0 (0.0)	1 (12.5)	6 (25.0)	0 (0.0)	
No change	2 (9.1)	4 (50.0)	3 (37.5)	5 (20.8)	8 (57.1)	
More	9 (40.9)	3 (37.5)	3 (37.5)	8 (33.3)	4 (28.6)	
Much more	4 (18.2)	0 (0.0)	0 (0.0)	3 (12.5)	1 (7.1)	
RED MEAT						
Much less	10 (45.5)	1 (12.5)	1 (12.5)	6 (25.0)	3 (21.4)	0.289
Less	6 (27.3)	2 (25.0)	0 (0.0)	5 (20.8)	2 (14.3)	
No change	3 (13.6)	3 (37.5)	4 (50.0)	6 (25.0)	6 (42.9)	
More	3 (13.6)	2 (25.0)	3 (37.5)	5 (20.8)	1 (7.1)	
Much more	0 (0.0)	0 (0.0)	0 (0.0)	2 (8.3)	2 (14.3)	
FRUITS						
Much less	2 (9.1)	1 (12.5)	1 (12.5)	2 (8.3)	1 (7.1)	0.312
Less	2 (9.1)	0 (0.0)	1 (12.5)	9 (37.5)	2 (14.3)	
No change	10 (45.5)	3 (37.5)	6 (75.0)	6 (25.0)	5 (35.7)	
More	4 (18.2)	3 (37.5)	0 (0.0)	6 (25.0)	4 (28.6)	
Much lore	4 (18.2)	1 (12.5)	0 (0.0)	1 (4.2)	2 (14.3)	
SOFT DRINKS						
Much less	4 (18.2)	2 (25.0)	0 (0.0)	3 (12.5)	1 (7.1)	0.561
Less	4 (18.2)	0 (0.0)	3 (37.5)	9 (37.5)	4 (28.6)	
No change	6 (27.3)	4 (50.0)	4 (50.0)	7 (29.2)	6 (42.9)	
More	6 (27.3)	1 (12.5)	1 (12.5)	5 (20.8)	3 (21.4)	
Much more	2 (9.1)	1 (12.5)	0 (0.0)	0 (0.0)	0 (0.0)	
DAIRY PRODUCTS						
Much less	5 (22.7)	1 (12.5)	0 (0.0)	1 (4.2)	3 (21.4)	0.436
Less	6 (27.3)	1 (12.5)	1 (12.5)	8 (33.3)	2 (14.3)	
No change	4 (18.2)	3 (37.5)	5 (62.5)	10 (41.7)	7 (50.0)	
More	6 (27.3)	2 (25.0)	1 (12.5)	2 (8.3)	1 (7.1)	
Much more	1 (4.5)	1 (12.5)	1 (12.5)	3 (12.5)	1 (7.1)	
FATTY-FRIED FOODS						
Much less	1 (4.5)	2 (25.0)	2 (25.0)	3 (12.5)	2 (14.3)	0.462
Less	6 (27.3)	1 (12.5)	1 (12.5)	8 (33.3)	1 (7.1)	
No change	4 (18.2)	1 (12.5)	4 (50.0)	7 (29.2)	5 (35.7)	
More	7 (31.8)	3 (37.5)	1 (12.5)	2 (8.3)	3 (21.4)	
Much more	4 (18.2)	1 (12.5)	0 (0.0)	4 (16.7)	3 (21.4)	
DESSERTS						
Much less	1 (4.5)	1 (12.5)	1 (12.5)	1 (4.2)	1 (7.1)	0.989
Less	4 (18.2)	2 (25.0)	1 (12.5)	6 (25.0)	2 (14.3)	
No change	6 (27.3)	2 (25.0)	2 (25.0)	9 (37.5)	5 (35.7)	
More	9 (40.9)	2 (25.0)	2 (25.0)	6 (25.0)	5 (35.7)	
Much more	2 (9.1)	1 (12.5)	2 (25.0)	2 (8.3)	1 (7.1)	
CANDY/SWEETS						
Much less	2 (9.1)	1 (12.5)	1 (12.5)	1 (4.2)	1 (7.1)	0.554
Less	4 (18.2)	3 (37.5)	0 (0.0)	8 (33.3)	2 (14.3)	
No change	7 (31.8)	2 (25.0)	6 (75.0)	5 (20.8)	5 (35.7)	
More	7 (31.8)	2 (25.0)	0 (0.0)	7 (29.2)	4 (28.6)	
Much more	2 (9.1)	0 (0.0)	1 (12.5)	3 (12.5)	2 (14.3)	
WHITE MEAT						
Much less	5 (22.7)	1 (12.5)	1 (12.5)	4 (16.7)	3 (21.4)	0.768
Less	5 (22.7)	2 (25.0)	1 (12.5)	4 (16.7)	2 (14.3)	
No change	3 (13.6)	3 (37.5)	4 (50.0)	7 (29.2)	5 (35.7)	
More	8 (36.4)	2 (25.0)	1 (12.5)	6 (25.0)	1 (7.1)	
Much more	1 (4.5)	0 (0.0)	1 (12.5)	3 (12.5)	3 (21.4)	

**Table 3:** Changes in cooking methods after arrival in Türkiye (n = 82).

Level of Change in Cooking Methods	Africa n (%)	Central Asia n (%)	Europe n (%)	Middle East n (%)	South/South East Asia n (%)	*p*
STIR-FRYING						
Much less	7(28.0)	1 (4.0)	2 (8.0)	9 (36.0)	6 (24.0)	0.911
Less	6 (40.0)	2 (13.3)	0 (0.0)	5 (33.3)	2 (13.3)	
No change	6 (24.0)	4 (16.0)	4 (16.0)	7 (28.0)	4 (16.0)	
More	2 (22.2)	1 (11.1)	2 (22.2)	2 (22.2)	2 (22.2)	
Much more	1 (50.0)	0 (0.0)	0 (0.0)	1 (50.0)	0 (0.0)	
DEEP-FRYING						
Much less	5 (21.7)	1 (4.3)	2 (8.7)	10 (43.5)	5 (21.7)	0.290
Less	6 (46.2)	3 (23.1)	0 (0.0)	2 (15.4)	2 (15.4)	
No change	7 (25.9)	3 (11.1)	6 (22.2)	7 (25.9)	4 (14.8)	
More	4 (36.4)	1 (9.1)	0 (0.0)	3 (27.3)	3 (27.3)	
Much more	0 (0.0)	0 (0.0)	0 (0.0)	2 (100)	0 (0.0)	
GRILLING						
Much less	7 (31.8)	2 (9.1)	2 (9.1)	9 (40.9)	2 (9.1)	0.113
Less	3 (17.6)	3 (17.6)	1 (5.9)	5 (29.4)	5 (29.4)	
No change	9 (31.0)	1 (3.4)	5 (17.2)	9 (31.0)	5 (17.2)	
More	0 (0.0)	2 (50.0)	0 (0.0)	0 (0.0)	2 (50.0)	
Much more	3 (75.0)	0 (0.0)	0 (0.0)	1 (25.0)	0 (0.0)	
BOILING						
Much less	4 (25.0)	2 (12.5)	1 (6.3)	5 (31.3)	4 (25.0)	0.717
Less	3 (30.0)	2 (20.0)	1 (10.0)	3 (30.0)	1 (10.0)	
No change	9 (26.5)	2 (5.9)	5 (14.7)	14 (41.2)	4 (11.8)	
More	4 (40.0)	2 (20.0)	1 (10.0)	0 (0.0)	3 (30.0)	
Much more	2 (33.3)	0 (0.0)	0 (0.0)	2 (33.3)	2 (33.3)	
MICROWAVE COOKING						
Much less	8 (38.1)	1 (4.8)	2 (9.5)	6 (28.6)	4 (19.0)	0.414
Less	3 (30.0)	1 (10.0)	0 (0.0)	5 (50.0)	1 (10.0)	
No change	9 (25.0)	3 (8.3)	4 (11.1)	12 (33.3)	8 (22.2)	
More	2 (25.0)	3 (37.5)	2 (25.0)	0 (0.0)	1 (12.5)	
Much more	0 (0.0)	0 (0.0)	0 (0.0)	1 (100)	0 (0.0)	

**Table 4:** Comparison of Culinary Acculturation Assessment Inventory pattern scores across sociodemographic characteristics.

Variables	The Basic Dietary Pattern (X ± SS)	The Meat-Oriented Pattern (X ± SS)	The Starch-Heavy Pattern (X ± SS)	The Accessory Foods Pattern (X ± SS)	The Culinary Practices Pattern (X ± SS)
GENDER					
Male (n = 49)	5.35 ± 1.05	4.57 ± 1.14	4.18 ± 1.33	4.39 ± 1.55	4.41 ± 1.39
Female (n = 33)	5.29 ± 1.08	4.09 ± 0.93	3.61 ± 0.84	4.57 ± 1.50	4.40 ± 1.03
*p*	0.799	0.048 *	0.031 *	0.595	0.983
REGION					
Africa (n = 22)	5.33 ± 1.06	4.57 ± 1.01	3.93 ± 1.40	4.24 ± 1.25	4.20 ± 1.49
Central Asia (n = 8)	5.75 ± 1.17	4.53 ± 0.87	4.10 ± 1.05	5.38 ± 0.86	4.25 ± 1.15
Europe (n = 8)	4.92 ± 1.35	4.38 ± 1.24	3.84 ± 1.39	4.56 ± 1.60	4.50 ± 1.38
Middle East (n = 24)	5.22 ± 0.88	4.17 ± 1.09	3.77 ± 0.86	4.64 ± 0.94	4.36 ± 1.29
South/South East Asia (n = 14)	5.26 ± 1.20	4.56 ± 1.37	4.37 ± 1.53	4.38 ± 1.61	4.52 ± 0.74
*p*	0.632	0.748	0.689	0.273	0.947
ECONOMIC STATUS					
Income less than expenses (n = 33)	5.30 ± 1.12	4.49 ± 0.90	4.14 ± 1.19	4.65 ± 1.19	4.32 ± 1.31
Income equals expenses (n = 40)	5.26 ± 1.06	4.23 ± 1.23	3.83 ± 1.25	4.37 ± 1.27	4.45 ± 1.18
Income exceeds expenses (n = 9)	5.54 ± 0.72	4.58 ± 1.05	3.89 ± 1.01	4.91 ± 1.35	4.30 ± 1.38
*p*	0.768	0.501	0.539	0.408	0.890
LENGTH OF STAY IN TÜRKİYE					
≤12 month (n = 58)	5.31 ± 1.11	4.58 ± 1.13	4.17 ± 1.28	4.55 ± 1.60	4.32 ± 1.33
13–36 month (n = 12)	5.39 ± 0.89	4.02 ± 1.04	3.65 ± 0.74	4.15 ± 1.57	4.39 ± 1.08
>36 month (n = 13)	5.32 ± 1.03	3.83 ± 0.57	3.28 ± 0.72	4.35 ± 1.11	4.81 ± 1.00
*p*	0.975	0.034 *	0.031	0.684	0.444
LANGUAGE PROFICIENCY					
Fluent (n = 14)	5.26 ± 1.12	4.11 ± 1.02	3.73 ± 0.99	4.63 ± 1.19	4.28 ± 1.21
Advanced (n = 24)	5.46 ± 1.13	4.55 ± 1.28	4.07 ± 1.36	4.51 ± 1.41	4.64 ± 1.03
Intermediate (n = 31)	5.20 ± 0.99	4.37 ± 0.98	3.94 ± 1.17	4.44 ± 1.12	4.14 ± 1.47
Beginner (n = 6)	5.67 ± 0.54	4.27 ± 0.86	4.31 ± 1.11	4.81 ± 1.18	4.17 ± 0.46
Unable to speak (n = 7)	4.99 ± 1.25	4.37 ± 1.28	3.86 ± 1.39	4.71 ± 1.66	4.97 ± 1.24
*p*	0.703	0.835	0.867	0.952	0.405
KNOWING A TURKISH INDIVIDUAL					
Yes	5.56 ± 1.07	4.38 ± 0.77	3.83 ± 0.48	4.77 ± 1.03	4.69 ± 0.78
No	5.27 ± 1.04	4.37 ± 1.13	3.98 ± 1.26	4.51 ± 1.28	4.34 ± 1.29
*p*	0.421	0.983	0.721	0.544	0.406
LIVING WITH A TURKISH INDIVIDUAL					
Yes	5.24 ± 1.05	4.12 ± 0.86	3.57 ± 0.96	4.33 ± 1.05	4.36 ± 1.35
No	5.34 ± 1.05	4.52 ± 1.18	4.19 ± 1.26	4.66 ± 1.34	4.40 ± 1.19
*p*	0.687	0.109	0.022 *	0.255	0.891

* *p* < 0.05.

## Data Availability

The datasets analyzed during the current study are not publicly available due to privacy and ethical restrictions but are available from the corresponding author on reasonable request.

## Supplementary Materials

The following supporting information can be downloaded at https://www.mdpi.com/article/10.3390/bs16050667/s1, Table S1: Dietary and meal preparation practices related to Turkish cuisine among international students, Table S2: Frequency of consumption of Turkish foods among international students.

## Abbreviations

The following abbreviations are used in this manuscript:

CAAI	Culinary Acculturation Assessment Inventory
AI	Artificial Intelligence
ANOVA	Analysis of Variance
SPSS	Statistical Package for the Social Sciences
χ^2^	Chi-Square Test
β	Standardized Regression Coefficient
B	Unstandardized Regression Coefficient
R^2^	Coefficient of Determination
SD	Standard Deviation
