# Supplementary material for: Culinary Acculturation Among International Students in Türkiye: Behavioral Insights and the Development of an AI-Supported Interactive Platform
Source: Behav Sci (Basel). 2026 Apr 28;16(5):667. doi: 10.3390/bs16050667 (PMC13203627; doi:10.3390/bs16050667)
Supplement: Supplementary file 1 [file behavsci-16-00667-s001.zip › behavsci-4245191-supplementary.pdf]

Supplementary Table S1. Dietary and meal preparation practices related to Turkish cuisine among international students (n = 82)

Habit / Practice	Never n (%)	⩽1 per month n (%)	2–3 per month n (%)	⩾2 - 3 per week n (%)	Everyday n (%)
Preparing Turkish Breakfast	19 (23.2)	17 (20.7)	15 (18.3)	16 (19.5)	12 (14.6)
Preparing Turkish Drinks (tea, etc.)	16 (19.5)	20 (24.4)	8 (9.8)	22 (26.8)	16 (19.5)
Cooking Turkish Dishes	28 (34.1)	16 (19.5)	9 (11.0)	29 (35.4)	5 (6.1)
Cooking with Oil	11 (13.4)	16 (19.5)	5 (6.1)	24 (29.3)	28 (34.1)
Cooking with Tomato Paste	12 (14.6)	12 (14.6)	8 (9.8)	28 (34.2)	22 (26.8)
Preparing Dishes with Yogurt/Ayran	27 (32.9)	17 (20.7)	5 (6.1)	30 (36.4)	8 (9.8)
Eating Together	7 (8.5)	22 (27.0)	15 (18.3)	33 (40.3)	19 (23.2)
Incorporating Turkish Cuisine into the Weekly Schedule	6 (7.3)	11 (13.4)	6 (7.3)	33 (40.3)	28 (34.1)
Regular Three-Meal Eating	4 (4.9)	24 (29.3)	9 (11.0)	30 (36.6)	24 (29.3)
Eating on the Floor	19 (23.2)	26 (31.7)	7 (8.5)	23 (28.0)	15 (18.3)

Supplementary Table S2. Frequency of consumption of Turkish foods among international students (n = 82).

Food Preference	Never n (%)	⩽1 per month n (%)	2–3 per month n (%)	⩾2 - 3 per week n (%)	Everyday n (%)
Bread/Flatbread/Plate	2 (2.4)	5 (6.1)	5 (6.1)	24 (29.3)	46 (56.1)
Pastry/Dumplings	4 (4.9)	13 (15.8)	18 (22.0)	38 (46.3)	9 (11.0)
Simit (Turkish bagel)	9 (11.0)	25 (30.5)	21 (25.6)	23 (28.0)	4 (4.9)
Rice/Bulgur/Pasta	0 (0.0)	2 (2.4)	9 (11.0)	38 (46.3)	33 (40.2)
Soup (lentil, tarhana, yogurt)	4 (4.9)	6 (7.3)	8 (9.8)	34 (41.5)	30 (36.6)
Kebab/Lahmacun/Doner	6 (7.3)	11 (13.4)	17 (20.7)	35 (42.7)	13 (15.9)

**Food Preference**	**Never n (%)**	**⩽1 per month n (%)**	**2–3 per month n (%)**	**⩾2 – 3 per week n (%)**	**Everyday n (%)**
Soupy Meat Dishes	6 (7.3)	15 (18.3)	18 (22.0)	32 (39.0)	11 (13.4)
Meatballs (Turkish cuisine)	7 (8.5)	25 (30.5)	22 (26.8)	25 (30.5)	3 (3.7)
Processed Meat Products (sausage, pastrami)	11(13.4)	19 (23.2)	18 (22.0)	30 (36.6)	4 (4.9)
Vegetable Dishes (with olive oil)	9 (11.0)	18 (22.0)	17 (20.7)	37 (45.1)	1 (1.2)
Salad / Appetizer	6 (7.3)	12 (14.6)	12 (14.6)	47 (57.3)	5 (6.1)
Pickles	14 (17.1)	22 (26.8)	11 (13.4)	34 (41.5)	1 (1.2)
Milk-based Desserts	7 (8.5)	19 (23.2)	15 (18.3)	32 (39.0)	9 (11.0)
Pastry Desserts (baklava)	3 (3.7)	21 (25.6)	16 (19.5)	35 (42.7)	7 (8.5)
Fruit Desserts / Compote	15 (18.3)	14 (17.1)	15 (18.3)	29 (35.4)	9 (11.0)
Turkish Delight / Confectionery	12 (14.6)	20 (24.4)	15 (18.3)	20 (24.4)	15 (18.3)
Tea	6 (7.3)	3 (3.7)	5 (6.1)	20 (24.4)	48 (58.5)
Turkish Coffee	21 (25.6)	18 (22.0)	7 (8.5)	16 (19.5)	20 (24.4)
Ayran / Kefir	6 (7.3)	3 (3.7)	10 (12.2)	36 (43.9)	27 (32.9)
Spices (red pepper, mint, sumac)	14 (17.1)	17 (20.7)	9 (11.0)	26 (31.7)	16 (19.5)
